# A High-Performance Circularly Polarized and Harmonic Rejection Rectenna for Electromagnetic Energy Harvesting

**DOI:** 10.3390/s23187725

**Published:** 2023-09-07

**Authors:** Zaed S. A. Abdulwali, Ali H. Alqahtani, Yosef T. Aladadi, Majeed A. S. Alkanhal, Yahya M. Al-Moliki, Khaled Aljaloud, Mohammed Thamer Alresheedi

**Affiliations:** 1 Department of Electrical Engineering, King Saud University, Riyadh 11421, Saudi Arabia; zabdulwali@ksu.edu.sa (Z.S.A.A.); yaladadi@ksu.edu.sa (Y.T.A.); majeed@ksu.edu.sa (M.A.S.A.); yalmoliki@ksu.edu.sa (Y.M.A.-M.); malresheedi@ksu.edu.sa (M.T.A.); 2 Department of Applied Electrical Engineering, Al-Muzahimya Campus, College of Applied Engineering, King Saud University, Riyadh 11421, Saudi Arabia; kaljaloud@ksu.edu.sa

**Keywords:** RF energy harvesting, circularly polarized antenna, harmonic rejection, stable DC power

## Abstract

This paper presents a novel circularly polarized rectenna designed for efficient electromagnetic energy harvesting at the 2.45 GHz ISM band. A compact antenna structure is designed to achieve high performance in terms of radiation efficiency, axial ratio, directivity, effective area, and harmonic rejection over the entire bandwidth of the ISM frequency band. The optimized rectifier circuit enhances the RF harvested energy efficiency, with an AC-to-DC conversion efficiency ranging from 36% to 70% for low-level input power ranging from −10 dBm to 0 dBm. The stable output of DC power confirms the suitability of this design for various practical applications, including wireless sensor networks, energy harvesting power supplies, medical implants, and environmental monitoring systems. Experimental validation, which includes both the reflection coefficient and radiation patterns of the designed antenna, confirms the accuracy of the simulation. The study found that the proposed energy harvesting system has a high total efficiency ranging from 53% to 63% and is well-suited for low-power energy harvesting (0 dBm) from ambient electromagnetic radiation. The proposed circularly polarized rectenna is a competitive option for efficient electromagnetic energy harvesting, both as a standalone unit and in an array, due to its high performance, feasibility, and versatility in meeting various energy harvesting requirements. This makes it a promising and cost-effective solution for various wireless communication applications, offering great potential for efficient energy harvesting from ambient electromagnetic radiation.

## 1. Introduction

Electromagnetic (EM) energy harvesting has received a lot of attention for a long time. In the early 1900s, Nikola Tesla experimented with wirelessly transmitting power through microwaves. However, his work was largely left unimplemented, as his experiments were vastly ahead of their time and the technology did not yet exist to make energy harvesting via microwaves feasible [1]. Advances in wireless technologies in the last few decades have made far-field energy harvesting a useful technology that has numerous applications and benefits, such as the Internet of Things (IoT), RF identification (RFID), wireless sensor networks (WSNs), and more [2,3,4,5].

**Figure 1:**
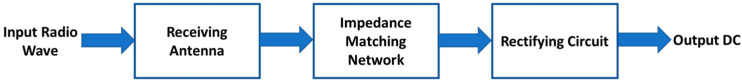
Block diagram of RF energy harvesting system.

Electromagnetic energy harvesting relies heavily on rectifying antennas (rectennas), which can convert RF energy to DC power. The low-pass filter (LPF), diodes, and DC pass capacitor are the main components of the standard rectenna, as shown in the block diagram of Figure 1 [3]. The task of RF power harvesting is performed by converting sent/ambient RF electromagnetic waves into an AC signal through the receiving antenna. This AC signal is then rectified into DC which can be stored in batteries or used directly to drive a specific circuit.

The greatest challenge in this system is the nonlinearity of the rectifier circuit, which has some drawbacks. On the one hand, the rectifier conversion efficiency from AC to DC depends on the input power. In other words, the greater the AC input power available at the rectifier, the higher the conversion efficiency that can be achieved, up to a certain point [6]. This issue can be mitigated by improving the performance of the pre-rectifying stages (i.e., the receiving antenna and matching circuit). Therefore, if a proper lossless matching network is used to match the antenna impedance to the rectifier impedance, maximum power transfer is achieved [4]. Consequently, the received antenna is the main part that improves the system’s efficiency. The antenna structure should have a high conversion efficiency to convert RF power into AC power [7,8]. Furthermore, the antenna should have a higher capturing efficiency that enables it to capture sufficient RF power to drive the rectifier circuit into the interested region. Increasing capturing efficiency means having a higher radiation efficiency, higher directivity, a lower load mismatching factor, and a lower polarization mismatching factor [9]. In [10], a dual-purpose radial-array rectenna was proposed for RF-energy harvesting IoT sensors, significantly enhancing RF energy capturing from a 360° region. It also enables precise orientation sensing using 5.8 GHz antennas, showcasing its versatility and potential for various applications.

On the other hand, nonlinear rectifying circuits, such as diodes, produce harmonics of the fundamental frequency. The unwanted harmonics impair system performance and result in harmonic interference that reduces the efficiency of the antenna and nearby circuit due to the coupling effect. As a result, a low-pass filter (LPF) is needed to suppress the harmonics to prevent harmonic interference, power re-radiation, and noise with antennas and nearby circuits [11]. However, the LPF will have insertion losses and increase the size and cost of the system. To elevate this effect, filters and microstrip antennas are usually fabricated together on the same substrate to improve cost and efficiency [12]. However, designing an antenna that does not resonate at the second and third resonance frequencies (i.e., also called a filtenna) would be the optimum solution [11]. Additionally, a wide-bandwidth antenna is also preferred, but it comes with decreasing efficiency. Furthermore, the effective bandwidth of the antenna will be limited by the lowest bandwidth of other energy-harvesting circuitry, such as rectifiers, that suffer from decreasing efficiency with increased bandwidth [7,13]. Moreover, improving the mismatching polarization factor makes circularly polarized antennas more preferable to linear polarization for some electromagnetic energy harvesting applications, especially due to the independence of the rotating angle and fading resistance [14].

Therefore, researchers focus on obtaining a competitive design that satisfies the aforementioned aspects or those required by different applications of ambient RF energy harvesting. The antenna used in such systems can be of any type, yet microstrip patch antennas are widely used due to their economical and electromechanical advantages [9,15,16,17,18]. However, lower bandwidth, directivity, and efficiency are the main drawbacks of such antennas [19]. Various techniques can be employed to overcome the disadvantages of antenna systems. For instance, using multiple antenna elements in an array can enhance the directivity of the system. However, this comes at the cost of increasing the size of the receiving antenna, which is dependent on the number of elements used. As a result, there is an increasing amount of research being dedicated to the development of compact and highly efficient antenna elements, which can enable the realization of high-performance antenna arrays in a small form factor [14,20,21,22]. Therefore, an antenna structure that combines harmonic rejection, higher directivity, and efficiency could be a good candidate for low-cost and compact energy harvesting.

In the existing literature, numerous designs have been proposed for electromagnetic energy harvesting applications. However, it is important to note that no single design can be considered perfect or universally suitable for all applications in this field. In [23], compact circularly polarized filtennas at 2.45 GHz are presented, but they have radiation efficiencies of less than 60%. In [24], the authors design a broadband antenna for energy harvesting, but its RF to DC efficiency is less than 55%, and the implementation complexity is high since the filter is printed on another layer. The authors of [25] proposed a highly efficient cross-dipole antenna with a reflector and filter integrated on the same substrate, achieving a high efficiency of 90%. However, the antenna size was very large, approaching the wavelength of the electromagnetic wave.

In [6], two-layer, two-port antennas were used to improve the collected power by obtaining dual linear polarization, but the size and cost of the antenna increased due to the need for two LPF filters, a matching circuit, and a rectifier. In [26], the focus was on minimizing the antenna structures (with maximum dimensions approaching a quarter wavelength) for energy harvesting applications. Although the design achieved a gain of 4.6 dBi and was very compact, it was a linearly polarized antenna with a very narrow bandwidth of 0.55%. In [27], a circularly polarized antenna with a gain of 6 dBi was presented, but the matching and axial ratio (AR) bandwidths were very small at 1.7% and 0.68%, respectively. Additionally, the antenna could not reject harmonics. In [28], a circularly polarized antenna was designed for use in RFID applications with a gain of 6.9 dB, but its bandwidth was limited to 1.5% at 2.45 GHz.

Metamaterial-based energy harvesting structures have also garnered significant attention from researchers due to their unique properties. The ability to engineer a metasurface and metamaterial surface with negative permeability and permittivity has opened up novel applications in various frequency bands, including energy harvesting [29], perfect lensing [30], and perfect absorption [31]. For instance, the metamaterial’s integration in the antenna design in [32] leads to a significant enhancement in the antenna’s gain across all frequency bands. This notable improvement solidifies the antenna as a promising solution for high-performance wireless communication systems. Moreover, a compact metamaterial-inspired antenna (MIA) enables efficient WiFi energy harvesting without complex networks, producing a rectified DC voltage for powering distributed microsystems [33]. In a related study [34], a dual-band metasurface simplifies design and facilitates high-efficiency electromagnetic energy harvesting at Wi-Fi frequencies, making it suitable for applications such as wireless power transfer. Additionally, an efficient miniaturized metasurface achieves over 78% conversion efficiency at 5.54 GHz, making it ideal for compact wireless sensor networks with wide angles [35]. However, a significant limitation of metamaterials is their narrow bandwidth of operation due to the resonance frequencies of their structures. This bandwidth limitation, coupled with the cost of fabrication, presents a challenge for the practical implementation of metamaterial-based energy harvesting structures. Nonetheless, ongoing research in this area holds great promise for the development of efficient and cost-effective energy harvesting solutions for a wide range of applications.

This paper presents a highly effective area rectenna for the 2.45 GHz ISM band, achieving compactness, circular polarization, high efficiency, harmonic rejection, sufficient improved bandwidth covering the ISM band, and high gain compared to traditional patch antennas. By effectively combining these features in a balanced tradeoff, the design enhances efficiency and expands the potential applications for energy harvesting. The antenna design structure has a bandwidth ratio of 4.08%, a directivity of 7.2 dBi, and a radiation efficiency of 92.5%. Moreover, the total size of the antenna is around 0.5 × 0.5 wavelength. The antenna can reject the second and third harmonics, which means it can be used without an LPF. The structure is one port and one layer, making it a simple and low-cost design. An optimized rectifier is also presented to evaluate the total rectenna performance and meet the antenna requirements.

## 2. Rectenna Design

To build a rectenna system, the most important parameters to evaluate the system should be considered. One of these parameters is the received power by the antenna used in this system, which is directly related to the receiving antenna’s effective area as [9]

(1) $${P_{r}=A_{eff}W_{inc}}_{}$$

where $A_{eff}$ is the antenna effective area that can be found by Equation (2), and $W_{inc}$ is the incident power density of the plane wave that can also be given by Equation (3).

(2) $$A_{eff}={\tau \;\eta }_{rad}\frac{\lambda^{2}D}{4\pi }\;PLF$$

(3) $$W_{inc}=\frac{{\left|E_{i}\right|}^{2}}{2\eta_{0}}$$

where τ is the matching transmission coefficient of the loaded antenna, which can be given by Equation (4), $\eta_{rad}$ is the radiation efficiency of the antenna, λ is the wavelength of the incident wave, D is the antenna directivity, *PLF* is the polarization mismatching loss factor that is given by Equation (5), *E_i_* is the incident electric field intensity, and $\eta_{0}$ is the surrounding medium’s characteristic impedance, which is assumed to be air in this research, so $\eta_{0}$ = 120π Ω.

(4) $$\tau =\frac{4R_{A}R_{L}}{{\left|Z_{A}+Z_{L}\right|}^{2}}$$

(5) $$PLF={\left|a_{i}.a_{r}\right|}^{2},$$

where *R_A_* and *Z_A_* are the receiving antenna input resistance and impedance, respectively; *R_L_* and *Z_L_* are the antenna load resistance and impedance, respectively; $a_{i}$ and $a_{r}$ are the incident field’s instantaneous direction vector and the antenna polarization direction vector, respectively. It must be emphasized that ($W_{inc}$ times $\lambda^{2}D/(4\pi )$) is the maximum effective area used to find $P_{r}$ when there is no mismatching loss (i.e., τ=1) or polarization mismatching loss (i.e.,  PLF=1).

### 2.1. Antenna Geometry

**Figure 2:**
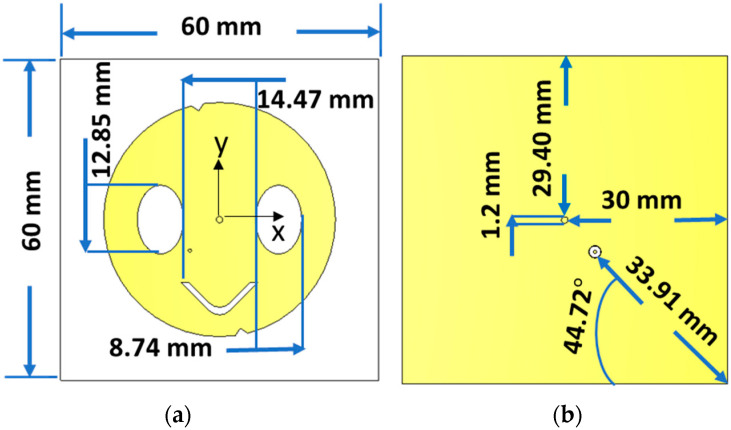
Structure of the proposed antenna, (**a**) the front side of the antenna, (**b**) the backside of the antenna.

The structure of a slotted circular patch antenna and its main important parameters are shown in Figure 2. The proposed antenna is a single-layer antenna consisting of a slotted circular patch on the front side, which is etched on a 60 mm × 60 mm Roger RT/duroid 5880 dielectric, a fully grounded reflector on the backside, a centered short via connecting the front and back sides, and coaxial probe feeding. The antenna shows the advantages of small size, high efficiency, directivity, and good matching in the 2.45 ISM band with circular polarization. In addition, it has harmonic rejection properties at the second and third harmonics. Thus, it is more suitable for power transfer and energy harvesting applications. This was achieved by minimizing the loss (i.e., increasing the radiation efficiency) and minimizing the rectenna size by self-suppressing the second and third harmonics.

Coaxial probe feeding was chosen because it has low transmission line loss and minimizes the area, thereby reducing the aforementioned problems. The feed position is chosen to achieve right-hand circular polarization.

The antenna is etched on a 60 mm × 60 mm (i.e., ~0.5 λ × 0.5 λ) Roger RT/duroid 5880 substrate with a dielectric constant (ε_r_ = 2.2) and electric tangent loss (tan(δ) = 0.0009), which are more suitable for enhancing the radiation efficiency. Although a low dielectric constant is not good for radiation characteristics, especially with a thin substrate [16], the proposed antenna has demonstrated good performance, as presented in the next section. The slotted edge is useful for obtaining this advantage since it causes the input impedance of the antenna to be constructively matched with the port impedance.

### 2.2. Rectifier Design

**Figure 3:**
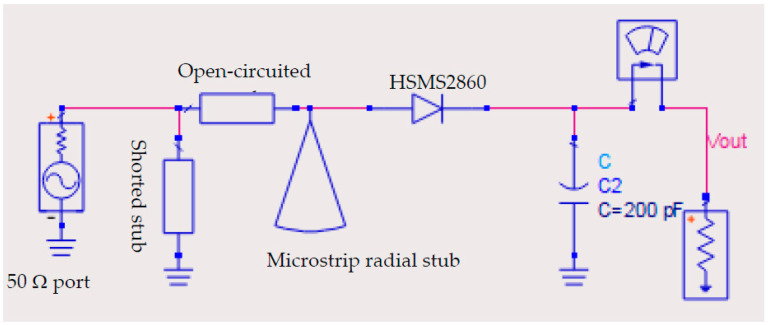
The rectifier circuit schematic with impedance matching circuit.

To build a full rectenna system, a rectification circuit was designed based on the antenna’s results. The rectifier circuit schematic built into ADS is shown in Figure 3. The goal was to maximize the DC output power at 1 KΩ, $P_{in}=-10\;to\;10$ dBm, and f=2.37−253 GHz.

The design of the rectifier circuit was based on optimizing the microstrip transmission line (impedance matching circuit) with different lengths and widths to achieve the desired goal. To ensure proper impedance matching, a Roger RT5880 substrate with a dielectric constant (ε_r_ = 2.2) and electric tangent loss (tan(δ) = 0.0009) was used.

To analyze the rectifier in the proposed rectenna system, the Harmonic Balance (HB) simulator was used due to the presence of the nonlinear Schottky diode (HSMS2860). HSMS2860 belongs to the HSMS-286x family of DC-biased detector diodes, which have been designed and optimized for use from 915 MHz to 5.8 GHz. The HSMS-286x family is ideal for RF/ID and RF Tag applications as well as large signal detection, modulation, RF to DC conversion, or voltage doubling. The electrical specification and spice parameter elements of this circuit are defined by the datasheet of the HSMS286x series in [36]. The circuit contains a series of transmission lines, a shorted stub, an open-circuited stub, and a microstrip radial stub between the 50 Ω port and the HSMS2860 diode.

To achieve optimal performance, a series HSMS 2860 Schottky diode was selected for its low turn-on voltage and fast switching speed at a frequency of operation of 2.45 GHz. Additionally, a shunt capacitor of 200 pF was chosen to appear as a short circuit for GHz frequencies and an open circuit for the rectified DC power.

Table 1 shows the optimized parameters of the impedance matching circuits, which were critical in achieving high AC-to-DC conversion efficiency ranging from 36% to 70% for low input power levels.

## 3. Simulation Results

The antenna port is excited by a Gaussian pulse with a 50 Ω probe feeding, propagating from the back of the antenna towards a positive *z*-axis direction, as depicted in Figure 2. To solve the electromagnetic problem with open boundaries (far-field problem), the finite integration technique (FIT) solver in Computer Simulation Technology (CST) is utilized. Furthermore, practical measurements are employed to validate the designed antenna and ensure its performance in real-world scenarios. In parallel, the performance evaluation of the proposed rectifier is carried out using the Harmonic Balance (HB) technique within the ADS full-wave simulator. This approach allows for a comprehensive analysis of the rectifier’s behavior and efficiency.

### 3.1. Antenna Reflection Coefficient and Input Impedance

**Figure 4:**
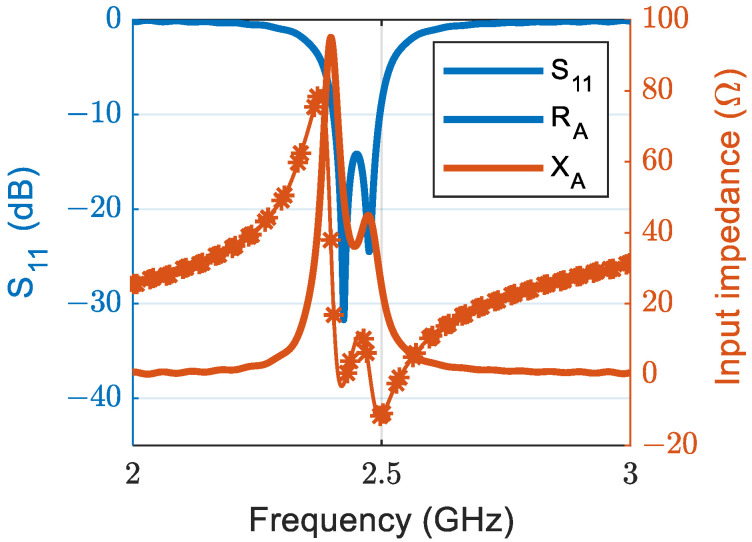
Return loss and input impedance of the final-designed antenna.

The reflection coefficient (S_11_) of a 50 Ω port as well as the input impedance of the antenna are shown in Figure 4. The antenna has a bandwidth of around 100 MHz with S_11_<−10 dB, which covers the whole ISM bandwidth.

The antenna design process is arranged into five stages. The first stage started by selecting the structure that would minimize the size and achieve good performance at the required bandwidth of 2.45 GHz in the ISM band. Thus, a probe-fed circular-patch microstrip antenna with Roger RT/duroid 5880 low permittivity and loss substrate material is first chosen based on recommendations in [17]. Then, using the design formula Equation (6) in [9], the radius of the circular patch, *r*, is calculated at the center resonance frequency, 2.45 GHz, using Roger RT/duroid 5880 material of a 2.2 permittivity (*ε_r_*) and 1.6 mm thickness (*h*). The radius calculation result of 23.13 mm is used to simulate the circular patch antenna using the full-wave simulator, where the feeding position and ground size are also chosen according to the recommendations of [9,17].

(6) $$r=\frac{F}{{\left\{1+\frac{2h}{\pi \varepsilon_{r}F}\;\left[\ln\left(\frac{\pi F}{2h}\right)+1.7726\right]\right\}}^{1/2}},\;F=\frac{8.791\times 10^{9}}{f_{r}\sqrt{\varepsilon_{r}}}$$

**Figure 5:**
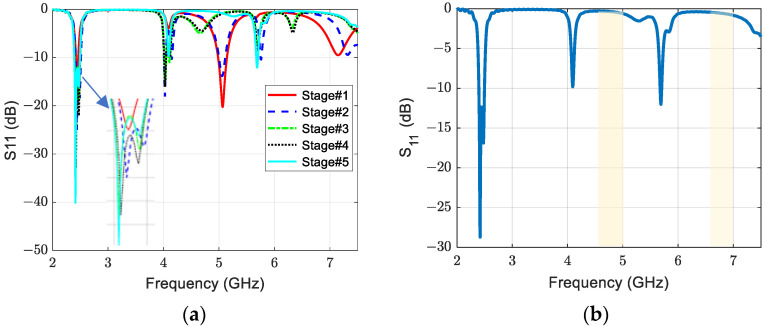
The reflection coefficient of (**a**) five design process stages and (**b**) final optimized stage.

Figure 5a shows the circular structure with the S_11_ of the five design process stages. The first stage (stage #1) is to adjust the antenna size for the targeted ISM band without any perturbations. In this stage, the antenna has a resonating mode of around 2.45 GHz and also resonates around the second and third harmonics. Furthermore, the radiation in this case is linear, and the antenna bandwidth is only 42 MHz. Therefore, the following design process stages are to solve these challenges by allowing suitable current perturbations to change the linear polarization to circular, increase the bandwidth, and reject the second and third harmonics. To have circular polarization, two orthogonal modes are generated in the second stage by loading the antenna with two optimum symmetric oval slots, as shown in Figure 2, and locating the feeding at 45° from the slot axis. The symmetric slots also improve the bandwidth and help minimize the radius of the circular patch from 23.13 mm to 21.86 mm. The size and position of these slots are adjusted and optimized to achieve the desired bandwidth and polarization axial ratio. The achieved bandwidth is 100 MHz, as shown in Figure 5a (see stage #2 line). The third stage (stage #3) aims to reject the harmonic where the patch antenna is loaded with a V-slot on its center bottom side to reject the second and third harmonics. The symmetric position with optimized dimensions of the V-slot improves the harmonic rejection property. To finely adjust the axial ratio and improve the matching, two triangular slits were introduced in the fourth stage (stage #4). In the last stage (stage #5), a centered via is used to further suppress the second and third harmonic frequencies at the second and third resonances. Therefore, this structure works as a filtenna since it works as a stop-band filter for harmonics, as shown in Figure 5b. Harmonics can cause rectenna performance degradation as well as nearby circuitry degradation, as explained in the introduction. Accordingly, the antenna is suitable for energy harvesting applications. The most important performance parameters that affect the effective area of the antenna are discussed in the next section.

**Figure 6:**
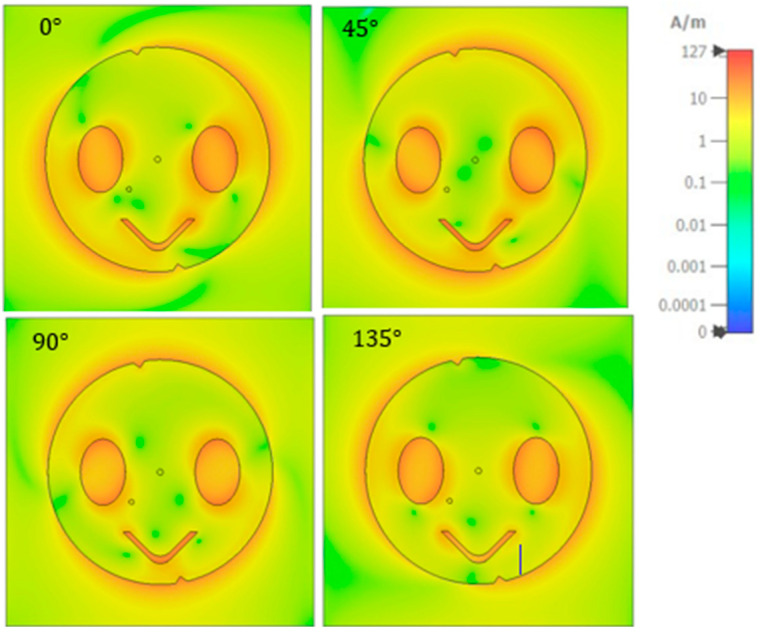
Current distribution of the proposed antenna at 2.45 GHz and different phases.

Figure 6 shows the current distribution of the proposed right-hand circularly polarized antenna at 2.45 GHz, which varies at different phases, indicating the counterclockwise movement of the surface current distribution at the edges. At all phases, the current distribution exhibits maximum amplitude at the symmetric oval slots, V-slot, and edges.

### 3.2. Antenna Radiation Efficiency and Axia Ratio

**Figure 7:**
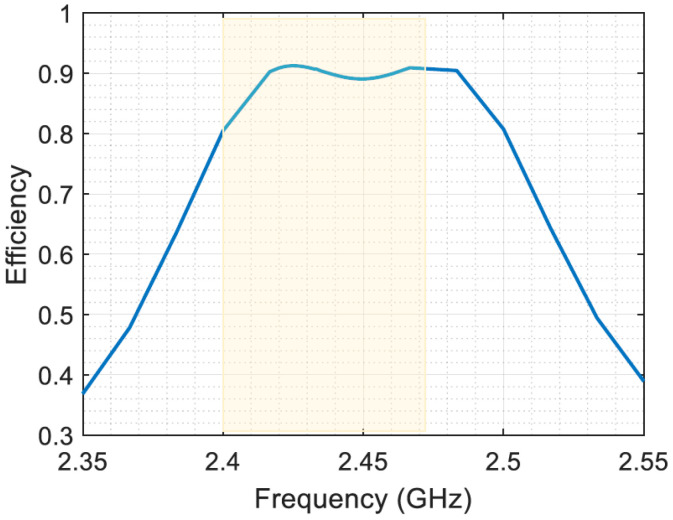
Antenna radiation efficiency.

The proposed antenna structure exhibits a radiation efficiency that varies between 80% and 91% over the frequency bandwidth of the ISM band, as shown in Figure 7. This is a significant achievement, as high radiation efficiency is critical for converting RF power into AC power for energy harvesting applications.

**Figure 8:**
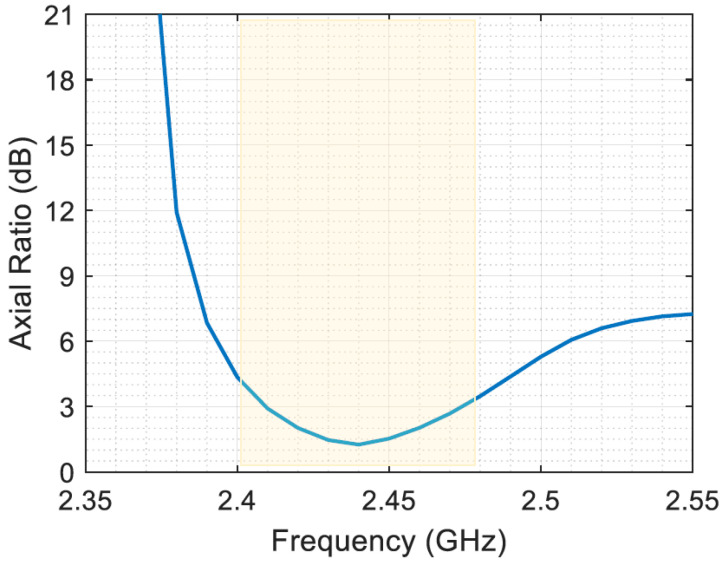
The axial ratio performance of a circularly polarized antenna.

Figure 8 shows the axial ratio performance of a circularly polarized antenna over a specific frequency range from 2.35 GHz to 2.55 GHz. The axial ratio, which is a measure of the polarization purity of an antenna, is plotted as a function of frequency, with the relevant frequency range highlighted for clarity. The results show that the proposed antenna is circularly polarized around the 2.45 GHz frequencies. The antenna exhibits outstanding circular polarization purity with an axial ratio consistently less than 3 dB. The fact that the axial ratio remains very small over the entire frequency range of interest is a testament to the antenna’s exceptional performance and its ability to maintain the desired polarization state with minimal distortion. Consequently, the amount of captured power will be increased to match the circular polarization and be half the amount for linear polarization ambient EM waves.

### 3.3. Antenna Directivity and Effective Area

**Figure 9:**
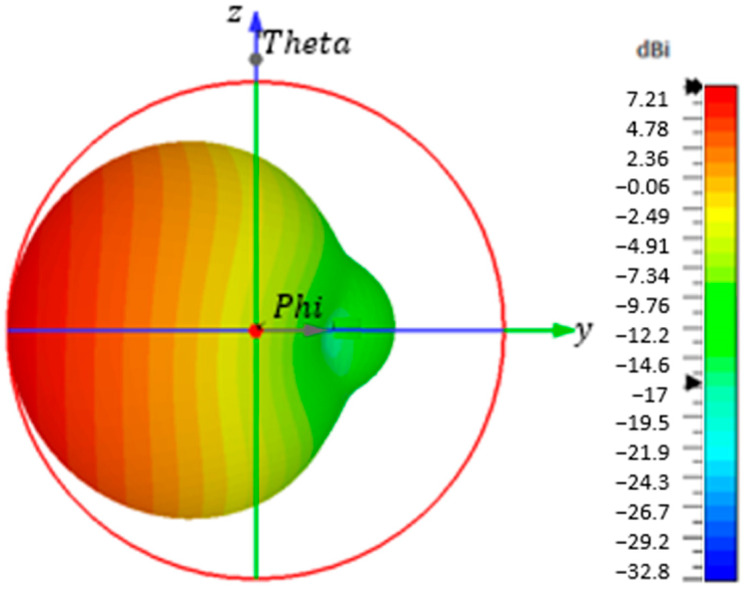
The antenna directivity pattern at 2.45 GHz.

**Figure 10:**
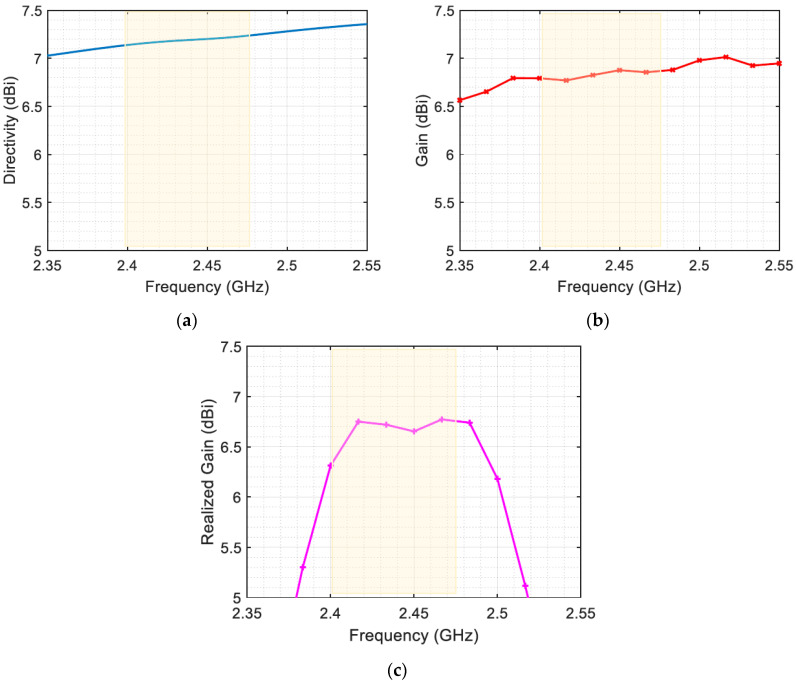
(**a**) Antenna directivity, (**b**) gain, and (**c**) realized gain vs. frequency.

The co-polarized directivity pattern of the proposed antenna at the studied frequency is shown in Figure 9. The maximum directivity is around 7.2 dBi in the main lobe of the center operating frequency, 2.45 GHz. The radiation pattern is unidirectional and symmetric in both the azimuth and elevation planes, with no significant side or back lobes. The plot of directivity with frequency variation in the same main lobe direction is shown in Figure 10a. The directivity of the antenna indicates its ability to concentrate radiation in a specific direction. However, directivity alone is not sufficient for a complete understanding of the antenna’s performance. To assess its effectiveness, it is important to consider the gain and realized gain as well. Gain refers to the ratio of the power radiated by the antenna in a specific direction to the power that an ideal isotropic antenna would radiate, assuming the same input power. It quantifies the antenna’s ability to efficiently radiate energy in a specific direction.

On the other hand, realized gain takes into account various factors such as losses, impedance matching, and efficiency. It provides a more realistic measure of the antenna’s performance in practical applications. Figure 10b,c present the antenna’s gain and realized gain, respectively.

In the context of energy harvesting, the effective area is a critical parameter, as it determines how much energy can be harvested from the ambient electromagnetic waves. Once the incident field is co-polarized with the antenna structure (i.e., PLF ≈ 1) and the connected load circuit is conjugate-matched (τ≈ 1), the maximum absorbed power is obtained. According to Equation (2), the effective area of the proposed antenna varies between 36 cm^2^ and 55 cm^2^ over the frequency range of 2.4 to 2.5 GHz.

A performance comparison between various published works and the proposed antenna is listed in Table 2. Among the listed designs, the proposed antenna exhibits a better trade-off between minimization, bandwidth, efficiency, and self-filtering for harmonic rejection (i.e., performing the LPF task). This makes it sufficient to drive the rectifier circuit for the targeted application with a low level of incident EM power.

### 3.4. AC–DC Efficiency

The efficiency of converting the received AC power to DC power is critical for energy harvesting applications. The AC-to-DC power conversion efficiency is calculated using Equation (7).

(7) $$\eta_{Ac-DC}=\frac{{V_{o}\times I}_{o}}{P_{in}}$$

where $P_{in}$ is the total time-average power coupled to the 50 Ω input port of the impedance matching network, and $V_{o}$ and $I_{o}$ are the output voltage and current on the load of the DC filter.

**Figure 11:**
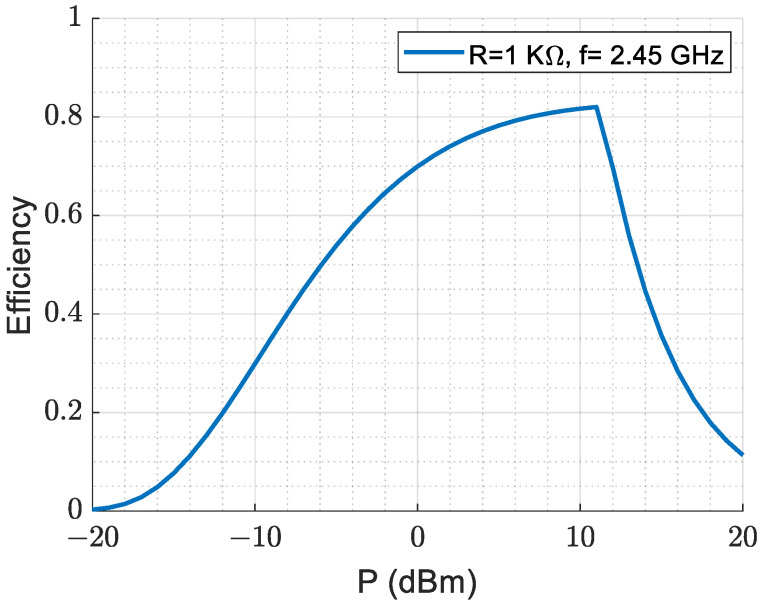
AC-to-DC radiation conversion efficiency.

Figure 11 illustrates the AC-to-DC conversion efficiency at 2.45 GHz with a 1 KΩ rectifier resistive load. The junction resistance in the equivalent circuit model of an HSMS 2860 Schottky diode [36] is dependent on the externally applied bias current, which makes AC-to-DC radiation conversion efficiency depend on the input power levels. The results show that for low input power levels ranging from −10 dBm to 0 dBm, the efficiency gradually increases from around 36% to 70%. The maximum DC power inversion occurs at an input AC power of 10 dBm. However, it is important to note that the performance of conversion efficiency deteriorates for higher input power levels (>11 dBm).

For practical wireless communication applications, the received input power levels are typically between −10 dBm and 0 dBm (an interesting region). Therefore, the proposed antenna structure with a rectifier circuit with AC-to-DC radiation conversion efficiency ranging from 36% to 70% for low input power levels is suitable for energy harvesting from wireless communication signals. The results demonstrate the advantages of the proposed rectenna for efficient energy harvesting from ambient electromagnetic radiation, which could be useful in various applications.

**Figure 12:**
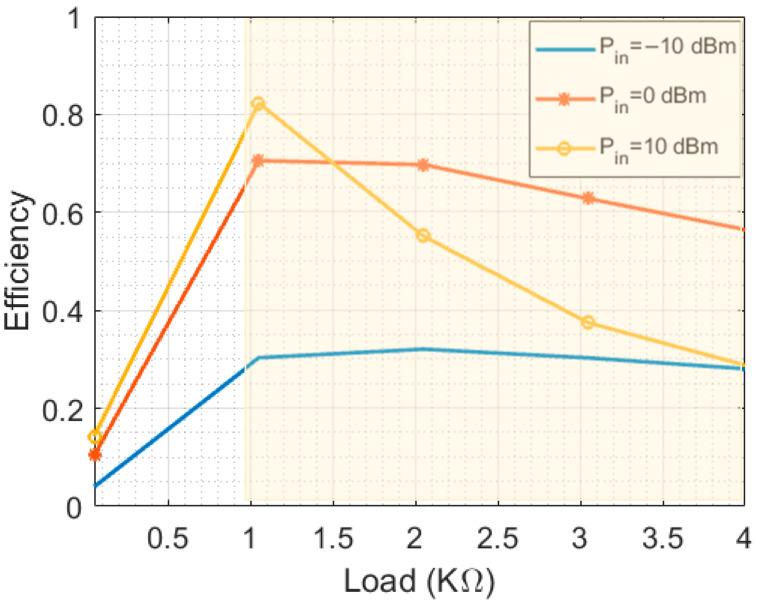
AC-to-DC radiation conversion efficiency at 2.45 GHz.

The overall efficiency of the energy harvesting system is affected by various factors, including the resistive load of the rectifier. Figure 12 shows the effect of changing the resistive load of the rectifier on the output DC power. Electromagnetic energy harvesting is utilized in various applications, including wireless sensor networks, RFID systems, energy harvesting power supplies, medical implants, and environmental monitoring systems. The input impedance of these applications varies, with wireless sensor networks typically ranging from 1 kΩ to 10 kΩ, RFID systems ranging from 50 Ω to 100 Ω, and others typically ranging from 1 kΩ to 100 kΩ [37]. The results of this work indicate that the DC power is stable in the range of 1 KΩ to 4 KΩ resistive load rectifiers for practical input AC power levels (−10 dB to 0 dB), which makes it a suitable candidate to support wireless sensor networks, energy harvesting power supplies, medical implants, and environmental monitoring systems.

**Figure 13:**
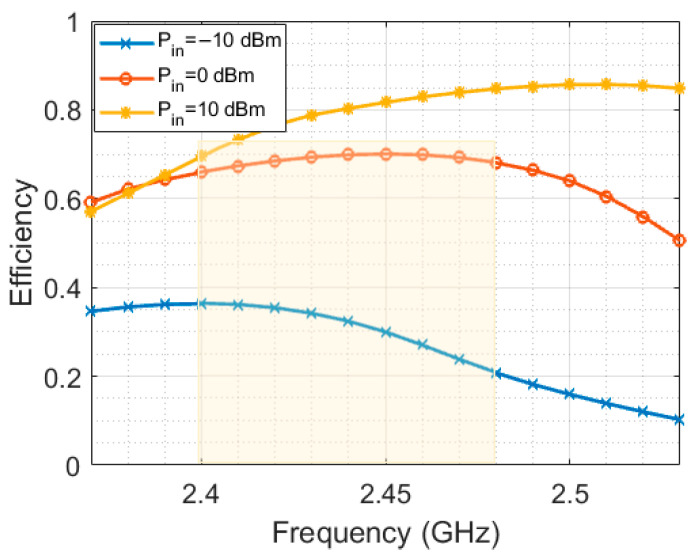
AC-to-DC radiation conversion efficiency at 1 KΩ resistive load.

In addition to the resistive load, the energy harvesting performance also varies with the input AC power level and operating frequency. Figure 13 shows the AC-to-DC conversion efficiency for frequencies in the ISM band at three different input AC power levels. The results indicate that the efficiency is within the range of 20% to 75% for the targeted bandwidth and different input power levels ranging from −10 dBm to 0 dBm.

**Figure 14:**
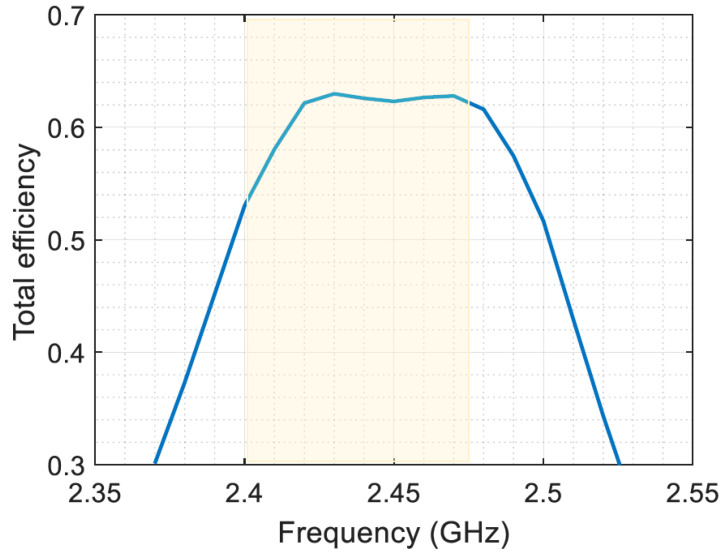
Total efficiency at 1 KΩ resistive load and 0 dBm input power.

Figure 14 presents the total efficiency of the energy harvesting system, which includes both AC-to-AC and AC-to-DC conversion efficiencies, plotted against frequency for the 0 dBm input power level and 1 KΩ resistive load. The results demonstrate that the proposed energy harvesting system offers a high total efficiency, ranging from 53% to 63%.

The high total efficiency of the energy harvesting system has important implications for various applications where low-power energy harvesting is essential. The results highlight the potential of the proposed antenna structure and energy harvesting system to provide a cost-effective and efficient energy harvesting solution for various wireless communication applications.

### 3.5. Experimental Validation

This section provides experimental validation of the antenna simulation results. A comparison is performed by analyzing the reflection coefficients and radiation patterns of both the simulated and measured data.

**Figure 15:**
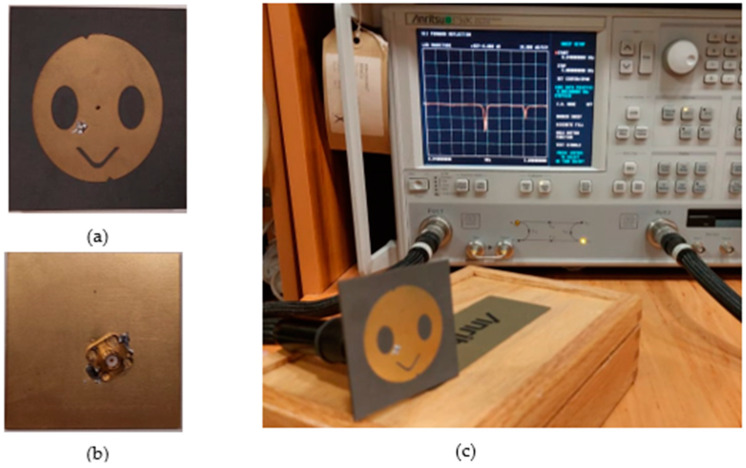
The fabricated antenna with vector network analyzer system. (**a**) The front side of the antenna, (**b**) the backside of the antenna, and (**c**) the vector network analyzer measurement system with the proposed antenna.

**Figure 16:**
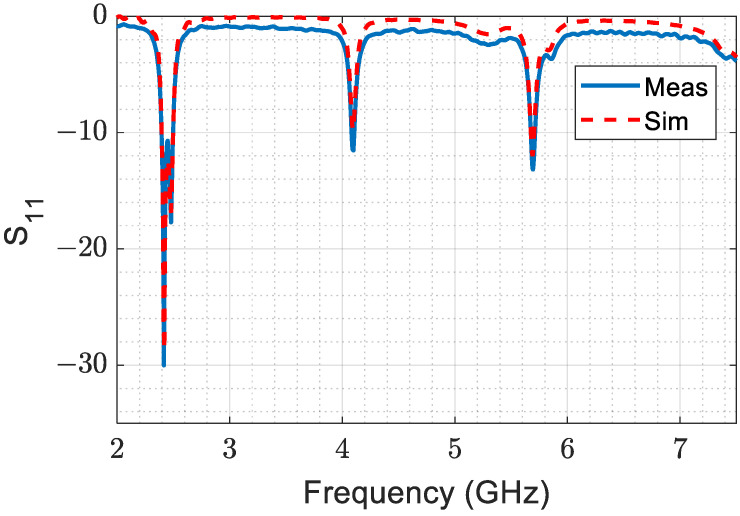
The measured and simulated reflection coefficients, S_11_.

The fabricated antenna and vector network analyzer system are shown in Figure 15. Figure 16 presents a comparison between the measured and simulated S_11_ values of the designed antenna, which indicates good agreement between the simulation and measurement results. This agreement confirms that the designed antenna is highly accurate and reliable.

**Figure 17:**
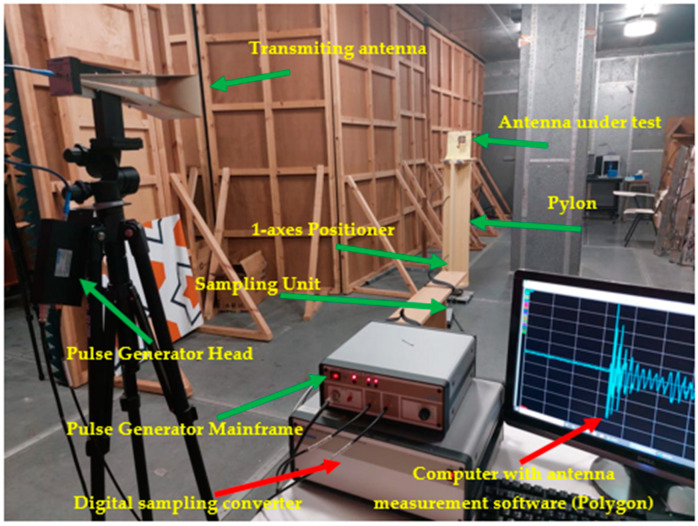
Geozondas time-domain antenna measurement setup.

**Figure 18:**
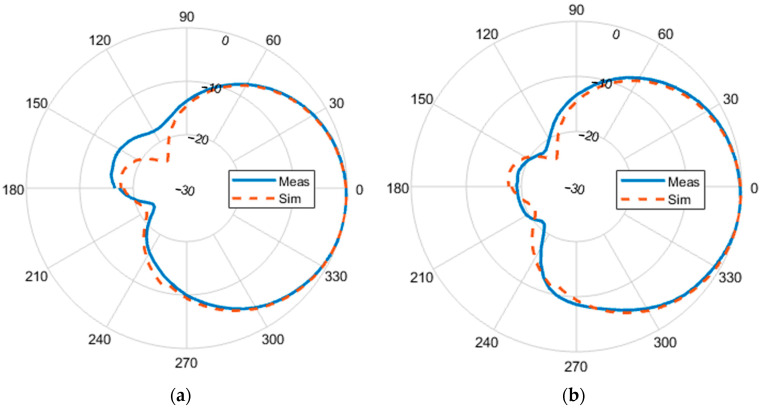
Normalized circularly polarized radiation patterns at 2.45 GHz: (**a**) azimuth and (**b**) elevation plane.

Figure 17 shows the Geozondas [38] time-domain antenna measurement setup used for measuring the radiation pattern of the designed antenna in the time domain. This setup consists of a pulsed signal generator, a digital sampling converter, a transmitting antenna, a receiving antenna, and an oscilloscope. The pulsed signal generator sends a short pulse to the transmitting antenna, which then radiates the pulse into space. The receiving antenna captures the signal, which is then analyzed by the oscilloscope to determine the receiving antenna’s radiation pattern. Figure 18a,b illustrates the measured and simulated polar radiation patterns of the designed antenna in the azimuth and elevation planes, respectively. The agreement between the simulation and measurement results demonstrates the accuracy and reliability of the designed antenna. The close match between the two sets of data in both the reflection coefficients and radiation patterns provides strong evidence to support the validity of the simulation approach and its ability to predict the behavior of the whole rectenna (antenna and rectifier) system.

**Figure 19:**
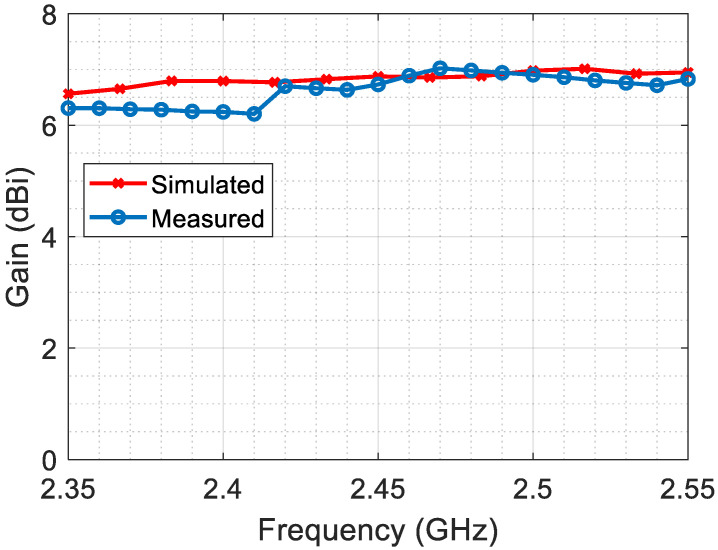
The measured gain compared to the simulation gain.

The gain is measured by calculating the performance of the proposed antenna in comparison to a reference antenna with a known gain. The two antennas are connected alternately to the same transmitter, and the received power is measured for each antenna. Both used antennas, the reference and the transmitter, are double-ridged horn antennas with lens GZ0126 DRH (see Figure 17). The gain of the proposed antenna is then determined by comparing the power levels. The measured gain of the proposed antenna is depicted in Figure 19, demonstrating an accepted agreement with the simulated gain.

## 4. Conclusions

This paper presents a circularly polarized rectenna designed for high-performance electromagnetic energy harvesting in the 2.45 GHz ISM frequency band. The proposed antenna structure achieves a radiation efficiency ranging from 80% to 91%, an effective area between 36 cm^2^ and 55 cm^2^, an axial ratio consistently less than 3 dB, and harmonic rejection over the bandwidth of the ISM band. Furthermore, it achieves a directivity of 7.2 dBi in the main lobe direction with negligible side/back lobes at the center frequency. The optimized rectifier enhances the RF harvested energy efficiency, with an AC-to-DC conversion efficiency ranging from 36% to 70% for input power levels ranging from −10 dBm to 0 dBm. The proposed design is feasible, versatile, and a competitive option for electromagnetic energy harvesting applications as a single element or in an array to meet various energy harvesting requirements. The stable output DC power in the range of 1 KΩ to 4 KΩ resistive load rectifiers for practical input AC power levels (−10 dB to 0 dB) confirms the suitability of this design for various applications, including wireless sensor networks, energy harvesting power supplies, medical implants, and environmental monitoring systems.

Additionally, the study finds that the proposed energy harvesting system has a high total efficiency that ranges from 53% to 63% at 0 dBm ambient electromagnetic power. Therefore, it is well suited for low-power energy harvesting, which has significant implications for various wireless communication applications.

The manufactured novel circularly polarized antenna was tested for its radiation pattern and S_11_ characteristics, with simulation and measurement results exhibiting remarkable agreement.

## Figures and Tables

**Table 1:** Simulation results.

Shorted Stub L_1_/W_1_	Open-Circuited Stub L_2_/W_2_	$\mathrm{Microstrip}\;\mathrm{Radial}\;\mathrm{Stub}\;L_{3}/W_{3}/\theta$ _3_
7.71/8.67	4/0.1	8.73/0.707/78°

**Table 2:** Comparison of antenna performance with the literature.

Ref.	Dimensions (𝛌)	Radiation Efficiency	Relative Bandwidth	Directivity	LPF Feature
[13]	0.49 × 0.49 × 0.0136	45%	5.3%	6.7 dBi	No
[24]	0.49 × 0.49 × 0.0136	50%	5.3%	6.9 dBi	Yes
[27]	0.46 × 0.46 × 0.03	89%	1.5%	6.98 dBi	-
[26]	0.474 × 0.474 × 0.0158	86%	1.7%	6.8 dBi	-
This work	0.49 × 0.49 × 0.0136	92%	4.08%	7.2 dBi	Yes

## Data Availability

Data sharing not applicable.
